# A Longitudinal Study on Mental Well-Being, Perceived Stress Level and Job-Related Meaningfulness of Austrian Telephone Emergency Service Counselors during the COVID-19 Pandemic

**DOI:** 10.3390/ijerph19063166

**Published:** 2022-03-08

**Authors:** Elke Humer, Christoph Pieh, Ida-Maria Kisler, Wolfgang Schimböck, Petra Schadenhofer

**Affiliations:** 1 Department for Psychotherapy and Biopsychosocial Health, University for Continuing Education Krems, 3500 Krems, Austria; christoph.pieh@donau-uni.ac.at; 2 ABILE-Viktor Frankl Education Austria, 3390 Melk, Austria; dr.i.kisler@gmail.com (I.-M.K.); wolfgang.schimboeck@liwest.at (W.S.); p.schadenhofer@kirche.at (P.S.); 3 Telephone Emergency Service—Lower Austria (TelefonSeelsorge NÖ), Diocese St. Pölten, 3100 St. Pölten, Austria

**Keywords:** COVID-19, counseling, helpline, job-related meaningfulness, stress, mental well-being

## Abstract

Telephone emergency service (TES) consultants have been challenged even more since the beginning of the pandemic. How the COVID-19 situation and the associated increasing demand for TES services affect the well-being and stress of TES counselors has not been assessed so far. This longitudinal study examined mental well-being (WHO-5), perceived stress level (PSS-10), and experienced job-related meaningfulness (CERES) of TES counselors at two measurement points during the pandemic. From December 2020 to January 2021 (t1), N = 374 counselors were recruited within the Austrian nationwide organization “TelefonSeelsorge”. From those, N = 108 also participated one year later at t2. Neither well-being nor perceived stress differed significantly between t1 and t2. There was a decrease in job-related meaningfulness (from 5.46 at t1 to 5.34 at t2; *p* < 0.001). The consultants identified loneliness and mental health as the most common problems of helpline callers at both measurement points. The results confirm a stable level of stress and well-being during the pandemic in TES consultants. However, they also show a slight decrease in perceived job-related meaningfulness. Well-being of counselors should be watched closely, as they are an important part of the psychosocial healthcare system.

## 1. Introduction

The COVID-19 pandemic and associated lockdown measures markedly impact most aspects of daily life, including economy, education, healthcare, mental health, and domestic violence [1]. This public health crisis has been reported to be associated with an increase in depression and other mental health issues [2,3], especially in the youth [4,5,6]. Higher psychological distress in healthcare workers compared to those working outside the healthcare industry has been reported at the beginning of the COVID-19 pandemic [7]. In the Austrian general population, an increase in mental health symptoms with the prolongation of the COVID-19 pandemic has been reported [8]. Thus, sufficient provision of mental healthcare services is required to mitigate the acute and longer-term mental health effects of the COVID-19 pandemic.

Telephone emergency services (TES) are an important part of the psychosocial healthcare system [9], providing readily available, confidential, and free-of-charge support in any state of emotional crisis (emergency number 142) [10,11]. The Austrian TES, named “TelefonSeelsorge“, was founded in 1966 to provide strictly anonymous, free-of-charge support that is available at all times, e.g. through counseling and stabilizing in difficult life situations with a special focus on suicide prevention. The services are mainly provided by trained paraprofessional volunteer counselors. Such nonclinical telephone emergency services are believed to provide essential emotional support services during, as well as in the aftermath of, the COVID-19 pandemic [9,12].

We previously assessed the mental well-being and perceived stress level of TES counselors, as well as the main topics of helpline callers at the end of the first year of the COVID-19 pandemic in Austria [12]. In brief, 374 counselors of the Austrian nationwide organization TelefonSeelsorge filled out an online survey conducted during the second wave of COVID-19 infections in Austria (December 2020 to January 2021). Counselors experienced higher mental well-being and less stress compared to a representative reference group of the Austrian general population [8]. Compared to pre-pandemic times, not only did the total number of calls increase, but also the topics of helpline callers changed. In particular, the topics loneliness, mental health, professional activities, and relationships were reported to be thematized more often since the emergence of the COVID-19 pandemic. Counselors reported in free-text questions that additional advanced training in the field of mental disorders and additional supervision would be helpful to support their work at TES [12].

It has been suggested that the increased demand for mental health services augments the risk for burnout among mental health practitioners during COVID-19 [13]. Moreover, the uncertainty and unpredictability of the COVID-19 pandemic is suggested to enhance stress level and mental health issues of the general population, but also of healthcare providers [13,14,15]. Experienced job-related meaningfulness is recognized as an important resource to mitigate the negative effects of occupational stress on mental well-being in healthcare personnel [16,17,18]. However, whether the ongoing coronavirus pandemic is accompanied with changes in mental well-being, stress levels, and job-related meaningfulness of counselors of TES has not been evaluated to date. As an enhanced perceived stress level decreases the capability of counselors to deliver optimal healthcare services [11,19], it is important to evaluate whether TES counselors experience a decline in mental health with the prolongation of the COVID-19 pandemic. Thus, monitoring counselors’ mental health in the face of the global pandemic is needed to enable timely intervention to address potential problems. Most previous studies on psychological distress in healthcare workers were conducted cross-sectionally, thus providing solely a snapshot of mental health at a single point in the pandemic. Only a few studies have measured psychological distress in healthcare workers repeatedly [20], and studies conducted at more than a single time point in helpline counselors are not available at all so far. Thus, to increase the understanding of the longer-term impact of the COVID-19 pandemic on telephone crisis intervention, Austrian TES counselors were surveyed again one year after the first survey, i.e., during the fourth wave of COVID-19 infections in Austria (November 2021 to January 2022).

The current study aims to examine the mental well-being, perceived stress level, and job-related meaningfulness of Austrian TES counselors. As the increased demand on helpline counselors (i.e., increase in the number of calls, change in the topics of helpline callers) might be an additional stressor and affect their well-being, a further aim was to assess potential changes in the topics of helpline calls with the prolongation of the pandemic.

To expand previous research, the following two research questions (RQs) were examined in the current study:

RQ 1: Do well-being, stress-level, and job-related meaningfulness in Austrian counselors of the TES change with the prolongation of the COVID-19 pandemic?
RQ 2: Do the topics thematized by Austrian TES helpline callers change with the ongoing COVID-19 pandemic?

## 2. Materials and Methods

### 2.1. Study Design

This quantitative longitudinal study comprised two online surveys conducted with the Research Electronic Data Capture (REDCap) platform (Vanderbilt University, Nashville, TN, USA) [21,22]. The “TelefonSeelsorge Österreich” informed their counselors (N = 856) about the study and provided the links to the surveys. Counselors were invited to take part in the first survey between 18 December 2020 and 24 January 2021 (t1). In total, 374 counselors completed the survey (response rate = 43.7%). The second survey was open from 15 November 2021 to 5 January 2022 (t2). To increase the response rate, the second survey was shorter (45 items) compared to the first one (73 items). Out of the 374 counselors who took part at t1, 108 counselors also completed the second survey (response rate = 28.9%). To enable matching of data of both surveys on an individual basis, counselors had to enter an 8-digit code at the start of each survey, which was generated by themselves following an instruction. The code consisted of the first and last letter of the given name of the mother, the father, and of the participant himself, followed by the day of birth of the mother.

Counselors’ participation was voluntary and they received no incentives. Counselors had to give electronic informed consent by agreeing to the data declaration to start the survey. The principles outlined by the Declaration of Helsinki were followed and the study was approved by the ethics committee of the University for Continuing Education Krems (Austria) with the following protocol code: EK GZ 35/2018-2021.

### 2.2. COVID-19 Situation in Austria

In Austria, the first COVID-19 cases were reported in February 2020, which was followed by a COVID-19 lockdown from the middle of March 2020 until the end of April 2020. After the nationwide lockdown, daily confirmed COVID-19 cases remained at a low level until summer 2020. With a strong increase in daily confirmed COVD-19 cases and hospitalization rates in autumn/winter 2020 (second wave of COVID-19 infections in Austria), further lockdown measures were introduced from the middle of November 2020 until the beginning of February 2021. During this time, the first survey of the current study was carried out (t1). After the openings on 8 February 2021, a third wave of infections hit Austria. Between May and July 2021, daily confirmed COVID-19 cases declined, and the vaccination rates increased significantly, which enabled a series of easing of COVID-19 restrictions. In late summer 2021, the “Delta” variant spread and the fourth wave of infections hit Austria. In view of the increasing number of hospitalized patients, new measures were introduced that increasingly restricted various areas of public life (such as shopping beyond basic needs, gastronomy, hairdressers, etc.) for unvaccinated people starting from the middle of November 2021. The steady increase in COVID-19 patients in intensive care units led to a new nationwide lockdown from 22 November 2021. At the same time, the government announced a general compulsory vaccination from 1 February 2022. The general lockdown ended in the middle of December, whereas the lockdown for unvaccinated people remained in place. In late December 2021, the “Omikron” variant spread and the fifth wave of infections emerged in Austria [23,24]. The second survey of the current study (t2) started with the lockdown of unvaccinated people (middle of November 2021) and lasted until the fifth wave of infections emerged (beginning of January 2022).

### 2.3. Measures

In accordance with the first survey, sociodemographic characteristics were surveyed (i.e., gender, age, education level, years in TES counseling, working time per month at TES).

#### 2.3.1. Well-Being (WHO-5)

Well-being was measured with the WHO-5 inventory [25,26]. It assesses well-being in the period of the previous past 2 weeks with five self-rating items on a six-point Likert scale from 0 = “at no time” to 5 = “all of the time”. The scales of the raw scores were multiplied by 4 to translate these health-related quality of life measures into a percentage scale from 0 (absence of well-being) to 100 (maximal well-being) [26]. Cronbach’s alpha for the WHO-5 for the first measuring point was α = 0.83 and α = 0.88 for the second measuring point.

#### 2.3.2. Perceived Stress (PSS-10)

The perceived stress level was measured with the Perceived Stress Scale-10 (PSS-10), a reliable and valid measure of the subjective stress level experienced over the previous month [27]. The PSS-10 comprises 10 items rated on a five-point Likert scale from 0 to 4. Four items need to be reverse scored before the total score of the PSS-10 is obtained by summing up all scores. Total scores range from 0 to 40, with a higher score indicating higher perceived stress. Cronbach’s alpha was α = 0.82 for t1 and α = 0.86 for t2.

#### 2.3.3. Job-Related Meaningfulness (CERES)

Experienced job-related meaningfulness was assessed with the Concern, Enthusiasm, Relevance, Efficacy, Satisfaction (CERES) scale [18]. The CERES consists of five key aspects of job-related meaningfulness rated on a 6-point Likert scale from 1 to 6. Items 2, 3, 4, and 5 are negatively worded and must be reverse coded before the total score of the CERES is obtained by summing up all scores and dividing them through five. Total scores range from 1 to 6, with a higher score indicating higher experienced job-related meaningfulness. Cronbach’s alpha was α = 0.73 for t1 and α = 0.80 for t2.

#### 2.3.4. Topics Thematized by Helpline Callers

Counselors of the TES were asked to rate the frequency of 15 different topics thematized by helpline callers on a six-point Likert scale from “1 = never” to “6 = always”. The surveyed 15 topics researched are explained in detail in our companion paper [12].

### 2.4. Statistics

Statistical analyses were conducted with the IBM SPSS (IBM Corporation, Armonk, NY, USA) Statistics 26 software program.

Chi-squared tests and *t*-tests for independent samples were conducted to investigate potential differences in the sociodemographic characteristics between counselors who responded or not to the second survey (responders vs. non-responders). Further t-tests were conducted to analyze potential differences in outcome variables (WHO-5, PSS-10, CERES) between responders and non-responders. The Bonferroni-corrected significance was set to *p* < 0.0167 (*p* < 0.05/3 *t*-tests). Differences in the topics of helpline callers between responders and non-responders were also investigated by *t*-tests, with the significance level set to *p* < 0.003 (*p* < 0.05/15 *t*-tests).

To evaluate potential differences in mental well-being, perceived stress, and job-related meaningfulness between the two time points, repeated-measures analyses of variance (RM-ANOVAs) were performed. The rating of mental health indices were the dependent variables. The time point (t1 vs. t2) was the within-subject factor. There were two between-subject factors: “gender” and “age”. The Greenhouse–Geisser-corrected values are presented. Bonferroni corrections were applied for the pairwise post hoc tests.

To evaluate potential differences in the topics thematized by helpline callers between the two time points, pairwise t-tests were performed, with the significance being set to *p* < 0.003 (*p* < 0.05/15 *t*-tests).

All statistical tests were performed two-tailed, and the significance level was set to *p* < 0.05 before Bonferroni correction.

## 3. Results

### 3.1. Participants

In total, out of 374 Austrian counselors of the TES who participated in the first online survey, 108 participated in the second survey (response rate = 28.9%). At the time of the first survey, responders were, on average, M = 57.26 (SD = 11.83) years old and did not differ from non-responders (M = 57.91, SD = 12.68); T (1, 372) = −0.387; *p* = 0.699. Responders were, on average, M = 7.79 (SD = 7.98) years in profession at t1, which also did not differ from non-responders (M = 8.65, SD = 8.79); T (1, 372) = −0.897; *p* = 0.380. The average working time at the TES at t1 was M = 16.55 (SD = 14.51) hours per month in responders and M = 15.19 (SD = 14.03) in non-responders; T (1, 372) = 0.840; *p* = 0.401. As summarized in Table 1, sociodemographic characteristics (gender, federal state, education) did not differ between responders and non-responders.

Well-being and perceived stress level did not differ between responders and non-responders at t1 (all *p*-values ≥ 0.053). Perceived job-related meaningfulness was slightly higher in responders (M = 5.48, SD = 0.585) compared to non-responders (M = 5.30, SD = 0.632); T (1, 372) = 2.508; *p* = 0.013. No differences in the reported frequency of different topics were reported at t1 between responders and non-responders (all *p*-values ≥ 0.32).

### 3.2. Results for RQ1

The counselors’ well-being and stress level did not differ between t1 and t2 (Table 2). The CERES was significantly lower at t2 compared to t1 (*p* < 0.001). No effect of gender was observed (all *p*-values ≥ 0.336). An effect of age was observed for the WHO-5 and CERES (*p* ≤ 0.01), showing higher mental well-being and perceived job-related meaningfulness with increasing age; however, the perceived stress level was not affected by age (*p* = 0.069). No interactions between time and age nor time and gender were observed on all investigated variables (all *p*-values ≥ 0.336).

### 3.3. Results for RQ2

**Figure 1:**
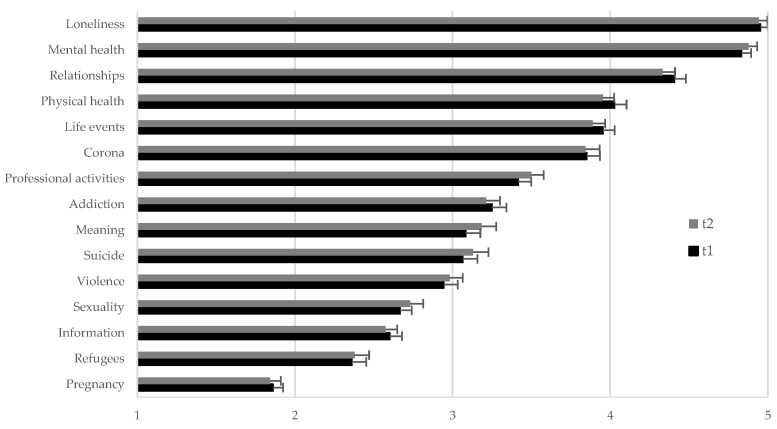
Counselors’ rating of the frequency of topics thematized by helpline callers during winter 2020/2021 (t1) vs. during winter 2021/2022 in Austria (t2) (N = 108). Note: counselors rated the frequency of each topic on a six-point scale from 1 = “never” to 6 = “always”. Data are shown as least squares means (LSM) ± standard error of the means.

As depicted in Figure 1, the frequency of different problems thematized by helpline callers did not differ between t2 and t1 (all *p*-values ≥ 0.057). At both time points, the topics loneliness and mental health were the most frequent topics of TES callers.

## 4. Discussion

The major aim of this study was to evaluate whether mental well-being, psychological distress, and experienced job-related meaningfulness in TES counselors changed with the prolongation of the COVID-19 pandemic in Austria. Results suggest that, while mental well-being and the perceived stress level in TES counselors remained unchanged at t2 vs. t1, their perceived job-related meaningfulness declined. Overall, it has to be noted that obtained values for CERES were still on a high level at t2 (5.34), being higher compared to Austrian nurses surveyed before the COVID-19 pandemic (4.97) [18]. To our best knowledge, there are no further reference values for the CERES available for healthcare workers. However, the higher CERES in TES counselors during the COVID-19 pandemic compared to nurses (even before the COVID-19 pandemic) corroborates the findings on mental well-being and psychological distress reported in our companion paper [12]. In more details, at t1, mental well-being (WHO-5) was higher and perceived stress level (PSS-10) was lower in TES counselors compared to the Austrian general population surveyed at the same time [8]. Thus, it seems that volunteer work at TES positively affects experienced meaningfulness and mental well-being, which might have some preventative effect against the development of psychological distress and mental health disorders during the COVID-19 crisis compared to the general population. Nevertheless, it is important to point out that TES counselors are not immune to developing mental health problems themselves. Enhanced symptoms of stress, burnout and mental health problems in telephone crisis support workers have been reported previously [11]. Therefore, results regarding the decrease in experienced job-related meaningfulness should not be undervalued. Moreover, contrary to the other investigated variables (well-being, stress level, topics of helpline callers), the subsample of counselors partaking at t2 does not seem to be representative for the larger sample of counselors who took part at t1 with respect to the perceived job-related meaningfulness. It rather seems that, predominantly, the counselors with a high degree of job-related meaningfulness took part in the second survey. As work-related perceptions of meaningfulness are considered as an important resource to reduce emotional exhaustion and maintain well-being at work [28], TES counselors should increase their mental hygiene during this stressful situation to prevent burnout and to be able to deliver high-quality crises intervention in the long term. Based on the responses to free-text questions on support wishes (data not shown), more exchange in the team, supervision and training, more appreciation of their work, and better technical equipment might help to maintain mental well-being of TES counselors in the long term.

The second aim of this study was to elucidate whether there are changes in the topics thematized by callers of the TES during the fourth wave of the COVID-19 pandemic in Austria (t2) compared to the second wave of the COVID-19 pandemic (t1). While the frequency of the different topics raised by helpline callers at t2 remained almost the same compared to t1, the quantity of helpline calls increased (personal information, TelefonSeelsorge Austria). During both time points, the topics loneliness and mental health were reported to be most often thematized by helpline callers. As reported in our previous paper, both topics have gained importance during the COVID-19 pandemic as compared to pre-pandemic times [12]. The predominance of the topics loneliness and mental health is strengthened by the general notion of enhanced mental health problems accompanying the COVID-19 pandemic [13,14,15], as well as previous studies suggesting that loneliness and social isolation are one of the main risk factors for mental disorders [29,30]. Overall, the increased quantity of calls, as well as the change in the topics of help seekers compared to pre-pandemic times, suggest that TES consultants have been challenged even more since the beginning of the pandemic.

When interpreting the results, the following limitations should be considered. First, the final sample size was rather small. Second, no reference values of mental health and job-related meaningfulness were available for the Austrian general population during the second survey. Moreover, a measurement point prior to the pandemic would have been needed to evaluate whether counselors’ well-being, stress level, and job-related meaningfulness were affected by this state of public health emergence *per se*. A further shortcoming is that the topics thematized by helpline callers were not assessed by objective data, but rather counselors’ self-reports.

## 5. Conclusions

The results confirm a stable level of stress and well-being during the pandemic in TES consultants. However, they also show a slight decrease in job-related meaningfulness. Well-being of counselors should be monitored closely to prevent emotional exhaustion and ensure optimal low-threshold crisis intervention during and in the aftermath of the pandemic.

## Figures and Tables

**Table 1:** Sociodemographic characteristics of the sample.

Characteristics	Responders (N = 108)		Non-Responders (N = 266)		Statistics
	N	%	N	%	
Gender					
Female	84	77.8	215	80.8	X^2^ (1, 374) = 0.445;
Male	24	22.2	51	19.2	*p* = 0.504
Federal state					
Burgenland	6	5.6	13	4.9	X^2^ (8, 374) = 13.83;
Lower Austria	14	13.0	26	9.8	*p* = 0.086
Vienna	17	15.7	46	17.3	
Carinthia	8	7.4	29	10.9	
Styria	5	4.6	33	12.4	
Upper Austria	16	14.8	25	9.4	
Salzburg	16	14.8	53	19.9	
Tyrol	9	8.3	19	7.1	
Vorarlberg	17	15.7	22	8.3	
Education					
Secondary school	2	1.9	5	1.9	X^2^ (4, 374) = 3.252;
Apprenticeship	9	8.3	24	9.0	*p* = 0.517
Vocational secondary school	24	22.2	45	16.9	
High School	18	16.7	64	24.1	
University	55	50.9	128	48.1	

Note: *p*-values (two-tailed), X^2^, chi-squared-test.

**Table 2:** Measures of mental well-being (WHO-5), perceived stress (PSS-10), and perceived job-related meaningfulness (CERES) in Austrian telephone emergency counselors in winter 2020/2021 (t1) vs. winter 2021/2022 (t2) and in female vs. male counselors.

		Time		Gender		Statistics
Characteristics		t1	t2	Female	Male	
						
WHO-5						
	N	108	108	84	24	time: F (1; 105) = 0.895; *p* = 0.346
	LSM	68.93	69.32	67.44	70.81	gender: F (1; 105) = 0.933; *p* = 0.336
	SEM	1.850	2.001	1.64	3.08	age: F (1; 105) = 6.769; *p* = 0.011
						
PSS-10						
	N	108	108	84	24	time F (1; 105) = 0.001; *p* = 0.977
	LSM	11.87	12.83	12.86	11.85	gender F (1; 105) = 0.790; *p* = 0.376
	SEM	0.594	0.687	0.536	1.01	age F (1; 105) = 3.369; *p* = 0.069
						
CERES						
	N	108	108	84	24	time F (1; 105) = 18.337; *p* < 0.001
	LSM	5.46	5.34	5.39	5.42	gender F (1; 105) = 0.039; *p* = 0.843
	SEM	0.068	0.081	0.063	0.119	age F (1; 105) = 12.645; *p* = 0.001

Note: *p*-values (two-tailed); LSM, least squares mean; SEM, standard error of the mean; F, F-test; WHO-5, well-being questionnaire of the World Health Organization (WHO); PSS-10, perceived stress scale 10; CERES, experienced job-related meaningfulness.

## Data Availability

The raw data supporting the conclusion of this article will be made available by the authors upon reasonable request.
